# Factors associated with early long-acting reversible contraceptives discontinuation in Ethiopia: evidence from the 2016 Ethiopian demographic and health survey

**DOI:** 10.1186/s13690-020-00419-w

**Published:** 2020-07-01

**Authors:** Gedefaw Abeje Fekadu, Akinyinka O. Omigbodun, Olumuyiwa A. Roberts, Alemayehu Worku Yalew

**Affiliations:** 1 Pan African University, Institute of Life and Earth Sciences (including Health and Agriculture), University of Ibadan, Ibadan, Nigeria; 2 College of Medicine and Health Sciences, School of Public Health, Bahir Dar University, Bahir Dar, Ethiopia; 3 University College Hospital, University of Ibadan, Ibadan, Nigeria; 4 School of Public health, College of Medicine, Addis Ababa University, Addis Ababa, Ethiopia

**Keywords:** IUD, Implant, Discontinuation, Long-acting, Ethiopia, Contraceptive use episode, Hazards ratio, Gompertz

## Abstract

**Background:**

Ethiopia is struggling to achieve the 2020 family planning target. But the current contraceptive prevalence uptake is low and dominated by short-acting methods. Contraceptive discontinuation rate is also high. This analysis was done to identify the reasons and factors associated with long-acting and reversible contraceptives (LARC) discontinuation in Ethiopia.

**Methods:**

The unit of analysis was LARC-use episodes in the 5 years preceding the survey, generated from the 2016 Ethiopian Demographic and Health Survey data. A total of 1385 LARC episodes were included. Data analysis was done using STATA 15. The event file generated from the contraceptive calendar was merged to the original data set to identify factors associated with LARC discontinuation. Univariate, bivariate and inferential analyses were done for 12 months LARC discontinuation.

**Result:**

Approximately 82% of LARC episodes were implants. About 45% of intrauterine device (IUD) and 61% of implant episodes were discontinued by 36 months. Side effects and the desire to become pregnant were the main reasons for discontinuation. Women aged 25–34 (HR = 0.26; 95% CI: 0.20–0.35) and those aged 35–49 (HR = 0.17; 95%CI: 0.11–0.26), women who participated in decision-making partially (HR = 0.53; 95%CI: 0.37–0.78), or fully (HR = 0.55; 95%CI: 0.40–0.74) and primiparous women (HR = 0.53, 95%CI: 0.33–0.86) had a lower hazard of discontinuing LARCs. On the other hand, women who had only primary education (HR = 1.32; 95%CI: 1.02–1.72) and women who were not sure about their fertility intention (HR = 2.11; 95%C: 1.28–3.46) had a higher likelihood of discontinuing these methods.

**Conclusion:**

Majority of LARC episodes were discontinued early, mainly due to the desire for pregnancy or experience of side effects. Older women, particularly those involved in household decision-making, and primipara were less likely to discontinue LARC. Women with only primary education and those uncertain about their fertility intention had a higher likelihood of discontinuation. Family planning service providers should focus on fertility intention and side effects when counseling women for contraceptive choice. Improving women’s participation in household decision-making may decrease LARC discontinuation in Ethiopia.

## Background

High contraceptive discontinuation rates for reasons other than the desire for pregnancy are public health problems and signal missed opportunities to promote and sustain contraceptive use [1–3]. Over one-third of the total unmet need for contraceptives and one-third of unintended pregnancies are due to contraceptive discontinuation [4, 5]. Although long-acting and reversible contraceptives (LARC) are less likely to be discontinued, the rate of discontinuation is high in Asia and Africa [6–9]. Nearly one-fifth of women in Pakistan, Nepal, Jordan, and India discontinued IUD within 1 year [10–13]. In Africa, 8.1% Nigerian women and 13.5% Egyptian women discontinued implanon at the end of 1 year [14, 15]. A facility-based study conducted in Debretabor town, Northwest Ethiopia, indicated that 65% of women discontinued implanon early [16]. Women mentioned method-related reasons like side effects and health concerns, desire to conceive, being sexually inactive and opposition from family as reasons for LARC discontinuation [7–9, 12, 13, 16–19]. Number of children, age of woman, history of contraceptive use, women’s occupation, husband occupation and educational status, experience of side-effects, post-insertion follow-up, economic status and poor counseling were risk factors for early LARC discontinuation [10, 12, 15, 16, 18, 20, 21].

The Ethiopian family planning program, like in other developing countries, is experiencing the leaking bucket phenomenon [22]. LARC use is low and coupled with early discontinuation. With this scenario, the country is less likely to achieve the 2020 national family planning target. Understanding the reasons and factors that affect LARC discontinuation is crucial to achieve the national target. More studies, with large sample sizes, are needed to identify the factors associated with LARC discontinuation in Ethiopia. Therefore, this analysis was done to estimate the LARC discontinuation rate and identify factors associated with it in Ethiopia.

## Methods

### Data

We used the 2016 EDHS data, the fourth DHS in Ethiopia, which was collected from January 18 to June 27, 2016 for this analysis. It is a community based, nationally representative, cross-sectional data collected from all 9 regions and two city administrations. For this study, the unit of analysis was episodes of LARC (IUD or implant) use among reproductive-age women. The LARC episode was generated from the EDHS monthly calendar data, which is month-by-month retrospective history of pregnancies, births, terminations, episodes of contraceptive use and reasons for discontinuation for the 5 years immediately before the survey. To get the LARC episodes, an event file was created from the calendar data following the steps mentioned in the DHS contraceptive calendar tutorial [23]. The event file contained a single record for each continuous episode including information when the episode was started, stopped, the duration of the episode, and what event preceded or followed the episode. In the EDHS, the contraceptive discontinuation rate is defined as, discontinuation among women aged 15–49 years who experienced an episode of contraceptive use within the 5 years preceding the survey. The percentage of episodes discontinued within 12, 24 or 36 months by reason for discontinuation, according to a specific method was calculated. Based on the definition, a woman may contribute more than one episode to the calculation. The calculation procedure is based on life table methods [24].

The contraceptive discontinuation risk analysis was for the 5 years before the survey. The period of observation was restricted to 3–62 months before the survey. That means episodes that occurred 3 months before the survey were excluded. This was done to avoid underestimation of contraceptive discontinuation due to method failure, as many women may not realize that they are pregnant in their first trimester. Based on these criteria, a total of 1385 LARC episodes were included in the analysis.

### Variables

#### Dependent variable

The outcome variable for this study was the duration of the risk period up to the occurrence of the contraceptive discontinuation. For this analysis, discontinuation was defined as stopping the use of long acting and reversible contraceptive methods (IUD or Implant). And early discontinuation was defined as stopping using IUD or implant before 3 or less years of insertion. The transition to non-user status was assessed at the first episode (or month) of non-use of any method. Computation of discontinuation rates was based on multi-decrement life tables from episodes 3 to 62 months prior to the survey. All episodes of implant and IUD use that began 1 month after the reference period and 3 months prior to the survey were used in this calculation.

#### Independent variables

The independent variables were grouped into socio-demographic (age at the end of episode, residence, educational status, partner), socio-economic (wealth index, working status, mobile phone ownership, internet use), fertility-related (parity and fertility preference), family planning program exposure (visit by family planning worker) and women participation in decision making.

Age is the woman’s age at the time of discontinuation (in years). This was generated by subtracting age at the time of interview from the time the event occurred. The residence was classified as urban and rural. The educational status had three categories: no formal education, primary and secondary or above. Wealth index was computed considering different household assets into 5 categories (poorest, poor, middle, rich and richest). In this analysis, we recoded the categories to 3 as poor, middle and rich. Working status and mobile phone use were used as coded in the EDHS data set. The internet use variable was recoded from three categories to two outcomes as “yes” and “no”.

Parity is the number of living children, classified into four categories: nullipara (no child), primipara (one child), multipara (two to 4 children) and grand multipara (five or more children). Fertility preference was assessed by asking women’s intention for future children at the time of the survey and had the following categories: intend to have, do not intend to have and undecided. Women participation in household decisions was computed based on three variables; decision-maker on women’s health, decision-maker on large household purchase and decision-maker to visit family. If the respondent decided alone or jointly with her husband on the particular issue, it was scored 1, otherwise 0 (zero). Summing all these scores, if the woman scored 3 out of 3, it was considered as she fully participated in household decision making, if she scored 2 out of 3, she was considered as “partially participated” and if she scored 0 or 1 considered as “not participated” in household decisions.

### Statistical analysis

Data management, univariate and bivariate analyses were performed using STATA 15. We employed the Gompertz proportional hazard model, a parametric model which estimates risk using likelihood procedures, to identify the effects of socio-economic, demographic and other factors on LARC discontinuation in Ethiopia. In this analysis, the hazard rate (ht) is the instantaneous risk of LARC discontinuation at month t, given that the LARC is still used just prior to that month. It is the limit of the number of LARC discontinuations per unit time divided by the number at risk as the time interval approaches zero. Hazard rates of LARC discontinuation are assumed to be a log-linear function of parameters for the effects of the independent variables. The hazard ratio was calculated at 12 months of discontinuation because this is the earliest time to discontinue these methods compared to the 24 and 36 months.

## Results

### LARC episodes by socio-demographic characteristics

**Fig. 1:**
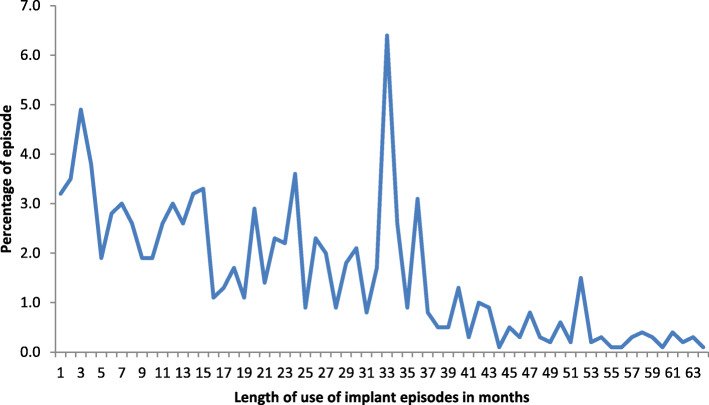
Percentage distribution of reported implant use episodes in Ethiopia, EDHS 2016

**Fig. 2:**
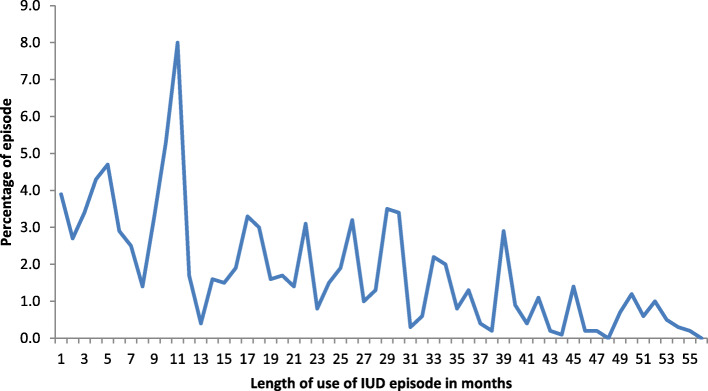
Percentage distribution of reported IUD use episodes in Ethiopia, EDHS 2016

A total of 1385 LARC (IUD and implant) episodes were observed in 5 years preceding the survey. Majority of the episodes (82%) were implants. The mean (standard deviation) duration of use for IUD was 21 (SD 16.4) months and 19.9 (SD 14.6) months for implant. Among implant users, the modal period of reported use was 33 months (Fig. 1). For IUD, the modal period of use was 10 months (Fig. 2).

About 90% of LARC episodes were among married or in-union women and nearly half of the episodes were among women aged 25–34. Similarly, about half of the episodes were among women with no formal education. Almost three-fourths of the LARC episodes were among rural women (Table 1).

**Table 1:** Contraceptive Use Episode by Selected Characteristics of Women in Ethiopia, EDHS 2016

Women characteristics	LARC type		
	IUD No. (%)	Implants No. (%)	All LARC No. (%)
Marital status			
Single	2 (0.9)	26 (2.3)	28 (2.0)
Married or living in union	231 (93.0)	1012 (89.0)	1243 (89.7)
Other (widowed or divorced)	15 (6.1)	99 (8.7)	114 (8.2)
Age at time of discontinuation (years)			
15 to 24	47 (19.1)	303 (26.6)	350 (25.3)
25 to 34	129 (52.1)	574 (50.5)	704 (50.8) 331 (23.9)
35 or more	71 (28.8)	259 (22.8)	
Place of residence			
Urban	100 (40.5)	273 (24.0)	374 (27.0)
Rural	148 (59.5)	863 (76.0)	1011 (73.0)
Education status			
No education	124 (50.1)	616 (54.2)	740 (53.5)
Primary	67 (26.9)	345 (30.3)	411 (29.7)
Secondary or above	57 (23.0)	176 (15.5)	233 (16.8)
Currently working			
No	154 (62.2)	682 (60.0)	836 (60.4)
Yes	94 (37.8)	455 (40.0)	548 (39.6)
Wealth index recoded			
Lowest	37 (14.9)	339 (29.9)	376 (27.2)
Middle	50 (20.3)	242 (21.3)	292 (21.1)
Rich	161 (64.8)	556 (48.9)	717 (51.7)
Participation in decision making			
Not participate	33 (13.4)	247 (21.7)	280 (20.2)
Partially participate	44 (17.6)	156 (13.8)	200 (14.5)
Fully participate	171 (69.0)	733 (64.5)	904 (65.3)
Owns a mobile telephone			
No	163 (65.6)	829 (73.0)	992 (71.7)
Yes	85 (34.4)	307 (27.0)	392 (28.3)
Fertility preference of the woman			
Have another	130 (52.4)	677 (59.5)	807 (58.3)
No more	113 (45.3)	408 (35.9)	520 (37.6)
Undecided	6 (2.3)	52 (4.6)	58 (4.2)
Internet use			
Never	229 (92.2)	1086 (95.6)	1315 (95.0)
Yes	19 (7.8)	50 (4.4)	70 (5.0)
Parity			
Nullipara	16 (6.3)	92 (8.1)	108 (7.8)
Primipara	35 (14.0)	223 (19.6)	258 (18.6)
Multipara	116 (46.8)	523 (46.0)	639 (46.2)
Grand multipara	82 (32.9)	298 (26.2)	380 (27.4)

### Discontinuation rate and reasons for discontinuation

Table 2 presented the LARC (IUD and Implant) discontinuation rate at three different times (12, 24 and 36 months) by reasons for discontinuation. Among episodes of LARC experienced within the 5 years preceding the survey, about 13% IUD and 11% of implant episodes were discontinued at the end of 12 months. Similarly, 26% IUD and 21.5% of implant episodes were discontinued at the end of 24 months. At the end of 3 years, about 45% of IUD and 61% of implant episodes were discontinued. Side effects or health concerns and desire to become pregnant were the main reasons for discontinuation of both methods across all time periods (Table 2).

**Table 2:** Twelve, 24 and 36 months LARC discontinuation rate by reason in Ethiopia, EDHS 2016

Reasons for discontinuation	Discontinuation rate					
	At 12 months		At 24 months		At 36 months	
	IUD	Implant	IUD	Implant	IUD	Implant
Become pregnant while using	0.0	0.3	0.4	0.3	0.4	0.3
Wanted to become pregnant	2.0	3.9	6.3	7.6	19.5	24.7
Other fertility related*	0.0	0.3	0.1	1.3	0.1	5.8
Side effects/health	7.0	2.5	10.5	6.7	10.5	12.8
Other method related**	2.1	2.2	4.5	2.9	10.1	4.7
Want more effective method	0.1	0.9	0.1	1.4	0.1	8.3
Other***	2.1	0.7	4.1	1.2	4.1	4.4
Total	13.3	10.9	26.0	21.5	44.8	61.1

* Marital dissolution, infrequent sex and menopause

** includes access, availability and convenient to use

*** Husband disapproval, difficult to get pregnant and fatalistic

The 12-month LARC discontinuation rate was higher among younger women (age 15–24) compared to older women (age 35 or more), and among women who reported a desire to have another child compared to women who do not want more children (Table 3).

**Table 3:** Twelve-month LARC Discontinuation Rate with Selected Characteristics of Women in Ethiopia, EDHS 2016

Characteristics	12-month LARC discontinuation rate (95%CI)
Marital status	
Single	27.0 (4.8–56.7)
Married or living in union	32.1 (27.3–36.9)
Other (widowed or divorced)	37.4 (20.2–54.7)
Age at time of discontinuation (years)	
15 to 24	36.7 (28.9–44.6)
25 to 34	32.4 (26.0–38.9)
35 or more	17.6 (9.6–27.5)
Place of residence	
Urban	24.1 (17.0–32.0)
Rural	32.0 (24.5–37.6)
Education status	
No education	28.6 (22.5–34.9)
Primary	30.7 (23.2–38.6)
Secondary or above	32.1 (19.8–45.0)
Currently working	
No	33.9 (27.8–40.0)
Yes	23.8 (17.4–30.7)
Wealth index	
Lowest	30.0 (18.1–42.8)
Middle	37.6 (28.2–47.0)
Rich	27.5 (22.0–33.2)
Participation in decision making	
Not participate	24.2 (14.9–34.8)
Partially participate	40.3 (25.8–54.4)
Fully participate	31.6 (26.2–37.2)
Owns a mobile telephone	
No	30.4 (25.1–35.9)
Yes	27.7 (20.0–35.9)
Fertility preference of the woman	
Have another	38.1 (32.1–44.1)
No more	15.6 (10.3–22.1)
Other	37.6 (17.5–57.8)
Ever used internet	
Never	30.2 (25.6–34.9)
Yes	23.6 (9.4–41.3)
Parity	
Nulliparous	40.6 (21.7–58.7)
Primipara	29.5 (19.5–29.5)
Multipara	33.3 (26.0–40.7)
Grand multipara	26.1 (18.4–34.4)

### Factors associated with 12-month LARC discontinuation

The analysis identified that mother’s age, educational status, parity, participation in household decision-making and fertility preference were significantly associated with 12-month LARC discontinuation. Compared to younger women, older women (age 25–34 (HR = 0.26; 95%C: 0.20–0.35) and age 35–39 (HR = 0.17; 95%C: 0.11–0.26)) had lower risks of discontinuing LARC. This analysis revealed that women who had only primary education had 32% higher (HR = 1.32; 95%C: 1.02–1.72) hazards of LARC discontinuation compared to women with no formal education. Women who partially (HR = 0.53; 95%C: 0.37–0.78) or fully (HR = 0.55; 95%C: 0.40–0.74) participated in decision making regarding household issues had lower hazards of LARC discontinuation compared to mothers who did not participate. Similarly, women who did not decide about their fertility intention had higher hazards (HR = 2.11; 95%C: 1.28–3.46) of discontinuing LARC compared to women who reported that they planned to have another child. In this analysis, primipara women had lower hazards (HR = 0.53, 95%CI: 0.33–0.86) of discontinuing LARC compared to nullipara (Table 4).

**Table 4:** Factors Associated with 12-month LARC Discontinuation among Women in Ethiopia, EDHS 2016

Characteristics	AHR (95%CI)
Age at time of discontinuation (years)	
15 to 24	Ref.
25 to 34	0.24 (0.18–0.32)***
35 or more	0.15 (0.10–0.23)***
Place of residence	
Urban	Ref.
Rural	1.24 (0.88–1.75)
Education status	
No education	Ref.
Primary	1.32 (1.02–1.71)*
Secondary or above	1.15 (0.76–1.76)
Partner education status	
No education	Ref.
Primary	1.25 (0.98–1.62)
Secondary or above	1.15 (0.76–1.75)
Currently working	
No	Ref.
Yes	0.88 (0.63–1.22)
Wealth index	
Lowest	Ref.
Middle	1.09 (0.79–1.49)
Rich	0.95 (0.70–1.28)
Participation in household decision making	
Not participate	Ref.
Partially participate	0.53 (0.37–0.77)***
Fully participate	0.54 (0.40–0.73)***
Owns a mobile telephone	
No	Ref.
Yes	1.42 (1.04–1.94)*
Fertility preference of the woman	
Have another	Ref.
No more	0.84 (0.63–1.11)
Undecided	1.90 (1.16–3.13)*
Ever used internet	
Never	Ref.
Yes	1.06 (0.62–1.83)
Parity	
Nullipara	Ref.
Primipara	0.53 (0.33–0.86)**
Multipara	0.86 (0.54–1.38)
Grand multipara	1.59 (0.90–2.82)

*Significant at 0.05

**Significant at 0.01

***Significant at <0.001

## Discussion

### Discontinuation rates

This community based national study demonstrated that there was high early LARC discontinuation rate. Although LARCs are intended to be used for a long time, the majority of the LARC episodes were discontinued early. Unless these contraceptives are retained for the intended period of time, the putative advantages of LARC will be in question. Implants are intended to be effective for up to 5 years but about two-thirds of the episodes were discontinued by the end of the third year. IUD can protect a woman from unintended pregnancy for up to 12 years but about half of IUD episodes had been discontinued by the end of the third year. The analysis revealed that the mean duration of IUD use was 21 months. With this scenario, it is difficult to think the health and economic returns of IUD in Ethiopia. Early discontinuation of these methods may expose women to unintended pregnancy. In addition, it reduces the dividends derivable from the investment made in these methods. These high early discontinuation rates may also signal problems in the implementation of family planning programmes, especially poor counseling before choice of contraception and provider-bias.

Although there is no optimal level for discontinuation rates, the 12, 24 and 36-month LARC discontinuation rates were all high. Such a high discontinuation rate, coupled with low LARC uptake, will affect the national effort to achieve the 2020 family planning target. The discontinuation rates in this study were similar to the pooled estimate of DHS data analysis from 21 low-income countries [25]. This suggests that the factors that affect LARC discontinuation in low-income countries may be similar in effect. Notably, more than half of the countries involved in the analysis were African countries where the barriers to LARC use tend to be similar.

The 12-month implant discontinuation rate in this study was higher than those found in Nigeria [14, 18] and Senegal [8] but similar to a report from Egypt [15]. The rate in this study was lower than that found in Dalo District, Southern Ethiopia [26], possibly due to differences in the population structure compared to the national pattern. Our study is based on a nationally representative sample. The 12-month IUD discontinuation rate in this survey was lower than the 25% rate found in India [11] and the 20% rate in Nepal [10], almost similar with those found in Jordan [13] and urban Senegal [8], but higher than findings in Nigeria [18]. The reason why the 12-month IUD discontinuation was higher in the study conducted in India may be the time change. The Indian study was based on the 2005/2006 NFHS data. Globally, many initiatives have been made to improve contraceptive use and decrease discontinuation rate. These, the time difference may explain the variation in these rates [27–29].

The discontinuation rates for both IUD and implants at 12 and 24 months were almost the same. This may be because both IUD and implant users experience a similar rate of side effects at this time. Our analysis identified side effects as the main reason for LARC discontinuation. This finding conforms to those of a systematic review done to estimate the LARC discontinuation rate among married women [17]. It however contrasts with a study conducted in urban Senegal, where the implants had a lower discontinuation rate than IUD [8]. The 12 and 24 month IUD and implant discontinuation rates here were lower than a report in Nigeria [18]. This may be due to the small sample size in the Nigerian study. When the number of study participants is small, the probability that it will contain typical users is high. In this case, the discontinuation rate may be lower.

The discontinuation rate for implants at 36 months was slightly higher compared to the discontinuation rate for IUD at 36 months. The reason for this is that implant users may prefer the implant to space pregnancy for a shorter period. These women may have a higher intention to become pregnant soon compared to IUD users. Therefore, they are more likely to discontinue early at 3 years compared to IUD users.

In this analysis, about 26% of IUD episodes were discontinued at the end of 24 months. This finding was similar to a study in Pakistan where the discontinuation rate was about 20% [30] and Vietnam (19.4%) [31] but lower than a study in India (34.5%) [20]. The time difference may be the reason for the lower discontinuation rate in this study compared to the Indian study, which was conducted in 2005/2006. The 24-month implant discontinuation rate was similar to a study in Nigeria (19.3%) [14] but lower than the study conducted in Debremarkos town (38.2%) [32]. The reason why the discontinuation rate was high in Debremarkos may be related to the age structure of women included in the Debremarkos study. Most women included in this study were a young age.

The study revealed that 45% IUD and 61% implant episodes were discontinued at the end of 36 months. The 36 month IUD discontinuation rate was higher than a study conducted in the USA (where the discontinuation rate was about 30%) [33] and Vietnam (26.9%) [31]. The reason for this might be better counseling for women in the USA compared to women in Ethiopia. Available pieces of evidence in Ethiopia identified that contraceptive counseling is poor; 18% family planning clients reported that it was difficult to understand what the provider discussed, there were deficiencies in the provision of information and communication and there was a shortage of materials [34, 35]. The difference from Vietnam may be the difference in the characteristics of women. The study in Vietnam included older women (aged up to 53 years) whose discontinuation risk is low. In addition, more women attended secondary education compared to women in the Ethiopian study. The 36-month implant discontinuation rate was higher than a study conducted in Mekele (38%) [36] but similar to the study conducted in Debretabor town (65%) [16]. The difference among these studies and the current study may be the small sample size included in the previous studies. In addition, the study in Mekele is discontinuation rate for 2 and half years. As time increases, the discontinuation rate is more likely to increase. In addition, these studies were institution-based and hence they may have underestimated the discontinuation rates.

#### Reasons for discontinuation

Intention to become pregnant, side effect or health concern and other fertility-related motives were the three commons reasons mentioned for LARC discontinuation in Ethiopia. This finding was similar to a DHS based pooled analysis from 21 low-income countries and meta-analysis [17, 37]. For both IUD and implant, the desire to become pregnant was the main reason for discontinuation. In addition, the mea duration of use (21 months), especially for IUD is very short. This may be due to poor counseling during contraceptive provision. Women who want to become pregnant after a short time may have used short-acting methods rather than using long-acting methods. The other reason might be provider bias. Family planning service providers may pressurize women to take long-acting methods rather than short-acting methods. In previous years, the Ethiopian government set an ambitious plan to increase the share of long-acting methods in the contraceptive method mix. Health providers were evaluated based on the number of long-acting contraceptives they provided. This might have biased family planning service providers to push women to use long-acting contraceptives without their interest. The authors understood that the Ministry of Health lifted these evaluation criteria by this time. Yet, it is important to inform family planning providers to follow the right based family planning approach rather than providing services based on provider targets. Side effects or health concerns were the second most common reasons for LARC discontinuation. This finding was in line with other studies [17, 18, 27, 32, 37]. LARCs have side effects like other drugs. But severe side effects are rare. The side effects or health concerns may be perceived or real. Women may associate other health conditions with contraceptive side effects due to biases and misconceptions. The side effects of LARC may not outweigh the problems women face due to unintended pregnancy. Therefore, health professionals should properly counsel women about common side effects of LARC and how to manage these side effects. Other fertility-related reasons (infrequent sex, menopause, and marital dissolution) were the third most common reasons for LARC discontinuation in Ethiopia. This finding was in line with other studies [11, 16, 37]. It is obvious that women who thought they are menopause stage or those who had infrequent sex due to various reasons are more likely to discontinue. Family service providers should take these into consideration when counseling women for contraceptives. These women can use short-acting methods.

#### Factors associated with LARC discontinuation

This analysis revealed that older women had a lower risk of LARC discontinuation. This finding was in line with studies conducted in Ethiopia and India [11, 20, 26, 38] but in contrast to a study in Pakistan. The reason for the difference among the finding in Pakistan may be the nature of the study participants. The participants in the Pakistani study were users of mobile outreach service programs. The reason why older women have a lower discontinuation rate may be that they have reached their desired fertility level. Hence, they are less likely to discontinue LARC early. On the other hand, younger women are more likely to desire more children. Therefore, they may discontinue LARC early if the barriers to have children are removed [20]. Health care providers should consider fertility intention when counseling young women for family planning.

Women who attended primary education had higher hazards of LARC discontinuation compared to women who did not attend formal education. This finding contradicts a study conducted in Debre Markos town [32] but in line with studies conducted in India which showed that women with little education were more likely to discontinue IUD than illiterate or better-educated women [11, 20]. Education is helpful to weigh the advantages and disadvantages of taking a particular action. It helps women to make the appropriate decision. But the primary education in Ethiopia may not be help women to reach this level of analysis.

Women who reported partial or full participation in household decision making had lower hazards of LARC discontinuation compared to mothers who did not participate. This study was in congruent with a study in Isfahan, Iran, which showed that women with higher household decision-making power in household affairs used contraception for a longer time compared to women whose husband made most of the household decisions [39]. It is understood that women who are involved in decision making on household issues are relatively empowered. Previous studies revealed that women empowerment had a positive impact on contraceptive use [40, 41]. Decision-making power among women helps them control over fertility and contraception [42]. A study in Uganda indicated that contraceptive use was 29% more likely in communities where women more commonly have unilateral control over household decisions [43]. A study in Eritrea identified that women’s final say in decisions regarding day-to-day household purchases was a significant predictor for contraception [44].

Women who did not decide about their fertility intention had higher hazards (HR = 2.11; 95%C: 1.28–3.46) to discontinue LARC compared to women who reported that they planned to have another child. The reason for this is that women who had the desire for more children are using the method for spacing. These women have a specific time in mind and took the LARC for the specified period. On the other hand, women whose fertility intention was undecided may discontinue the method any time as they did not have time in mind. Studies in Kenya revealed that women who desired no more children tended to use LAPM more than those wanting a child within or after some years as well as those uncertain about their future intentions [45, 46].

Primipara women had lower hazards to discontinue LARC compared to nulliparous women. This finding was in line with DHS based contraceptive discontinuation analysis of 21 low-income countries. The analyses showed that higher parity significantly reduces the hazards of contraceptive discontinuation [37]. Another study from India also revealed this fact; IUD discontinuation rates were decreasing with increasing parity [20]. The likelihood of experiencing IUD discontinuation was significantly lower among acceptor with two children at the time of use as compared to acceptors with no children [20]. The reason for this is that primipara women may be using contraceptives for spacing. Therefore, they may use these methods for a long time. The other possible reason is that primipara women may be relatively older compared to nulliparous women. As seen from this analysis, younger women are less likely to discontinue LARC compared to older women. Women with high parity might have achieved their desired family size while women at younger parity are likely to postpone childbearing for certain time.

This analysis was based on nationally representative large implant and IUD episodes. In addition, the data is more reliable as it is based on high quality interviewer training, data collection and supervision. However, the data may be affected by recall bias although women are more likely to remember events related to long ating method use. The other limitation of this study might be on the reasons of discontinuation. The calendar method considers only the main reason for discontinuation although the woman may have more than one reason to discontinue.

## Conclusion

LARC discontinuation rate was high in Ethiopia compared to the national targets to reduce unintended pregnancy and increase the contraceptive prevalence rate. Most IUD and implant episodes were discontinued early. The mean duration of IUD use 21 months, which is by far shorter than the protection IUD provided. The high LARC discontinuation rate, coupled with low uptake, is a big challenge to achieving the targeted long-acting contraceptive prevalence rate. Desire to become pregnant and side effects (health concerns) were the main reasons for discontinuation in most LARC episodes. Younger women, women who were not involved in household decision-making, nullipara women and women with primary education had a higher probability to discontinue LARC episodes. Women who were not sure about their fertility intention were also more likely to discontinue LARC compared to women who want more children. Therefore, family planning service providers should focus on the fertility intention of women and LARC side effects when counseling women for choice of contraceptives. We recommend that family planning service providers should clearly evaluate the fertility intention of young and nulliparous women before providing LARC. Encouraging women participation in household decision-making may also increase longer use of LARC episodes in Ethiopia.

## Data Availability

The dataset supporting the conclusion of this article is available in the demographic and health surveys Program repository at https://www.dhsprogram.com/data/dataset/Ethiopia_Standard-DHS_2016.cfm?flag=0.

## Abbreviations

CI: Confidence interval
DHS: Demographic and health survey
EDHS: Ethiopian demographic and health survey
HR: Hazard ratio
IUD: Intrauterine device
LARC: long-acting reversible contraceptive
SD: Standard deviation
USA: United States of America
